# A comparison of Chinese and non-Chinese Canadian patients hospitalized with heart failure

**DOI:** 10.1186/1471-2261-13-114

**Published:** 2013-12-10

**Authors:** Darwin F Yeung, Nicole K Van Dyke, Laura C Maclagan, Gordon W Moe, Baiju R Shah, Maria Chiu, Douglas S Lee, Dennis T Ko, Ching Lau, Jack V Tu

**Affiliations:** 1 Institute for Clinical Evaluative Sciences, 2075 Bayview Avenue G250, Toronto ON, Canada; 2 Faculty of Medicine, University of Toronto, Toronto, Canada; 3 St. Michael’s Hospital, Toronto, ON, Canada; 4 Division of Cardiology, University Health Network, Toronto ON, Canada; 5 Division of Cardiology, Schulich Heart Centre, Sunnybrook Health Sciences Centre, Toronto ON, Canada; 6 Division of Endocrinology, Sunnybrook Health Sciences Centre, Toronto ON, Canada

**Keywords:** Heart failure, Hypertension, Valvular heart disease

## Abstract

**Background:**

Canadians of Chinese descent, represent one of the fastest growing visible minority groups in Canada, (as well as the second largest), but relatively little is known about the clinical features of heart failure (HF) in Chinese-Canadian versus non-Chinese Canadian patients.

**Methods:**

We conducted a population-based analysis of urban patients hospitalized in Ontario, Canada for the first time with a most responsible diagnosis of HF between April 1, 1995 and March 31, 2008. Among the 99,278 patients, 1,339 (1.3%) were classified as Chinese using a previously validated list of Chinese surnames. Through linkage to other administrative databases, we compared the clinical characteristics, pharmacological management, and outcomes of Chinese versus non-Chinese HF patients.

**Results:**

Ischemic heart disease was identified as the possible etiology of HF in a greater proportion of non-Chinese patients (47.7% vs. 35.3%; p < 0.001) whereas hypertension (26.1% vs. 16.1%; p < 0.001) and valvular heart disease (11.6% vs. 7.2%; p < 0.001) were relatively more common in Chinese patients. Chinese patients were prescribed angiotensin-converting enzyme (ACE) inhibitors less frequently (57.5% vs. 66.4%, p < 0.001) and angiotensin receptor blockers (ARBs) more frequently (17.4% vs. 8.9%, p < 0.001) compared to non-Chinese patients. They were also less likely to be adherent to ACE inhibitors over a 1-year follow up period. However, the 1-year case-fatality rates were comparable between the Chinese (31.7%) and non-Chinese (30.2%) subjects (p = 0.24).

**Conclusion:**

There are important differences in the causes and medical management of HF in Chinese and non-Chinese patients residing in Canada. Despite these differences, the long-term outcomes of HF patients were similar.

## Background

Heart failure (HF) is a source of substantial mortality and morbidity. However, the impact of HF on specific ethnic populations has seldom been studied [1]. Chinese Canadians constitute 4.7% of the Ontario population and represent 21% of its visible minority [2] population and yet, little is known about the clinical features of HF in Chinese patients, relative to other Canadians. In a retrospective study of 404 Chinese patients and 1,129 white patients referred to an urban tertiary care cardiology clinic in Toronto, Canada, HF was less common in Chinese patients than in white patients [3]. Chinese patients diagnosed with HF generally exhibited higher left ventricular ejection fractions than white patients. In a cohort study involving six communities across the United States, Chinese participants demonstrated lower incidence rates of HF than African American, Hispanic, and white participants [4]. A previous study from Alberta demonstrated that Chinese heart failure patients in Alberta had higher adjusted 1-year mortality rates than South Asian or non-Asian patients [5]. Evaluating differences in the treatment and outcomes of HF among ethnic groups could potentially contribute to our understanding of disease processes and guide prevention and treatment strategies. This study compared the clinical characteristics, management and outcomes of Chinese and non-Chinese patients in a large population-based cohort of HF patients in Ontario, Canada. It builds upon previous studies which focused predominantly on incidence or mortality outcomes alone.

## Methods

### Study setting

Our cohort included patients who were hospitalized for the first time due to HF in Ontario, Canada between April 1, 1995 and March 31, 2008. As of 2006, Ontario accounted for 38.5% of the Canadian population with 12.0 million people. Individuals who identified themselves as Chinese constituted 4.7% of the Ontario population and represented one of the largest visible minority groups in the province, second to South Asians [2].

### Sources of data

Hospitalization data were obtained from the Canadian Institute for Health Information Discharge Abstract Database. Data on prescription drug use for patients 65 years of age or older were compiled from the Ontario Drug Benefit Database. Mortality data were determined through the Ontario Registered Persons Database. The immigration status of the study participants was determined through linkage to the Permanent Resident Database of Citizenship and Immigration Canada, with recent immigrants identified as those who immigrated to Ontario after 1984.

### Cohort construction

We identified patients from the Discharge Abstract Database using diagnosis codes from the *International Classification of Diseases, Ninth Revision*, *Clinical Modification* (ICD-9 CM) and the *Tenth Revision* (ICD-10). An audit of the database showed 96% accuracy in the coding of HF based on the Framingham diagnostic criteria [6]. A total of 292,733 patients were hospitalized with a most responsible diagnosis of HF (ICD-9 code 428 or ICD-10 code I50) between April 1, 1995 and March 31, 2008. We excluded patients who were not residents of Ontario (n = 2,606), lacked a valid Ontario health card number (n = 2,229), or were less than 20 or over 105 years of age (n = 828). We also excluded patients whose HF was listed as an in-hospital complication (n = 1,842). Patients transferred to another hospital were only counted once, with subsequent admissions (n = 6,705) linked to the index event. To identify first instances of HF requiring hospital admission, we excluded hospitalizations for HF that occurred up to five years prior to the index admission (n = 156,056). Hospitalizations occurring more than five years after the index admission and thus not representing the first admission in the study period were also excluded (n = 2,076). All patients hospitalized in rural hospitals (n = 21,113) were removed from the analysis due to the low number of Chinese patients in these settings.

### Classification by Chinese ethnicity

A previously validated list of 1,133 Chinese surnames was used to identify patients of Chinese ethnicity prior to anonymization of the hospital discharge data [7]. This list has been shown to have a sensitivity of 80.6% and a positive predictive value of 92% in classifying individuals who previously identified themselves as Chinese from primary data sources.

### Patient characteristics

Demographic information including age and sex were obtained for the Chinese and non-Chinese groups. Comorbid disease status was quantified using the Charlson-Deyo Comorbidity Index, a commonly used measure of comorbidity burden [8-10]. Prevalence of cardiovascular comorbidities not accounted for by the index such as atrial fibrillation or atrial flutter, hypertension, ischemic heart disease and valvular heart disease were also determined. Comorbid conditions included those coded in the secondary diagnosis fields of the index discharge abstract or in the primary or secondary diagnosis fields from the discharge abstracts of hospitalizations that occurred up to five years prior to the index event. Based on the cardiovascular disease history obtained from the discharge abstracts, each HF case was associated with an underlying etiology according to the hierarchy of (1) ischemic heart disease, (2) valvular heart disease, (3) hypertension, or (4) other/unknown diseases, resulting in four mutually exclusive groups [11].

### Pharmacotherapy

We linked our cohort to the Ontario Drug Benefit Database to determine the percentage of Chinese and non-Chinese patients 65 years of age or older who received at least one prescription for a given medication relevant to HF management within 90 days of discharge. Rates of medications filled within 90 days prior to the index admission were also determined for the two groups.

### Adherence

We compared the proportions of Chinese and non-Chinese patients demonstrating high adherence to ACE inhibitors, ARBs and beta-blockers. Adherence was estimated using data available from 1998 onwards from the Ontario Drug Benefit Database. Only patients who initiated therapy within 30 days of discharge were included in the analysis since they were the most likely to require the medication in the long-term. Adherence was measured by determining the proportion of days covered by the medication prescriptions within 6-month and 1-year time frames following discharge for the index HF hospitalization. The numerator was the number of days of medication supplied for each prescription after hospital discharge to six-months or 1-year after the discharge date. The denominator was the number of days between the date the first prescription was filled and six-months or 1-year after the discharge date. For patients who died before the 6-month or 1-year end-points, the proportion of days between the date the first prescription was filled and death for which a supply of the medication was available was determined instead. A high level of adherence was defined as ≥ 80% of days covered, a commonly used cut-off in clinical trials. A sensitivity analysis was also conducted excluding patients who were prescribed the medication within 90 days prior to admission in order to focus primarily on adherence in patients prescribed these medications for the first time.

### Case-fatality and readmission

We determined rates of case-fatality and readmission occurring 30 days or 1 year post-discharge. Readmission due primarily to HF was distinguished from all-cause readmission. A composite outcome, measuring the rate of either death or readmission for HF, was also determined. Case-fatality included death for any reason, while readmission represented at least one non-elective hospitalization after the first admission. The number of deaths occurring in our cohort was determined by linkage to the Ontario Registered Persons Database.

### Statistical analysis

All rates were reported as the number of events per 100 patients. Crude rates in the Chinese group were compared to those in the non-Chinese group using the chi-square test. Logistic regression modeling was used to adjust for patient level characteristics that might confound the comparison of clinical outcomes. The variables included in the risk-adjustment models were modified from previously developed models based on medical administrative claims in the United States. The mortality model included 23 candidate predictors for mortality associated with age, sex and co-morbid diseases [12]. The readmission model included 36 candidate predictors of readmission for HF patients [13] (see Additional file 1 for model covariates). The composite outcome of mortality or readmission was adjusted using variables from the mortality risk adjustment model. A model predictor was considered present if it was coded as a main, secondary or pre-admit diagnosis for any acute inpatient hospitalization in the past five years. Adjusted clinical outcomes for each group were considered to be significantly different from the rate of all patients if the overall crude rate did not fall within the 95% confidence limits of the adjusted rate. All statistical analyses and data linkages were conducted using SAS release 9.1.

The research was approved by the Research Ethics Board of Sunnybrook Health Sciences Centre.

## Results

### Clinical characteristics

We identified 99,278 patients residing in an urban area who were hospitalized in Ontario for the first time with a most responsible diagnosis of HF from April 1, 1995 to March 31, 2008. Of these patients, 1,339 (1.3%) were classified as Chinese based on their surname. The mean age of the Chinese group was 76.1 years compared to 75.9 years for the non-Chinese group (p = 0.22) (Table 1). Female patients accounted for 53.0% of the Chinese group and 51.2% of the non-Chinese group (p = 0.20). The mean Charlson-Deyo comorbidity index score was similar between the two groups (p = 0.10). Of note, Chinese patients demonstrated a higher prevalence of diabetes in general (23.5% vs. 20.0%, p < 0.001) and diabetes with chronic complications (14.0% vs. 10.1%, p < 0.001). Chronic obstructive pulmonary disease was markedly more common among non-Chinese patients compared to Chinese patients (22.3% vs. 13.4%, p < 0.001).

**Table 1:** Demographic and clinical characteristics of patients

	**Chinese (n = 1,339)**	**Non-Chinese (n = 97,939)**	***P***
Mean age, y	76.1	75.9	0.22
Female, %	53.0	51.2	0.20
Charlson-Deyo Comorbidity Index			
Mean score	1.85	1.77	0.10
Cardiovascular comorbidities, %			
Acute myocardial infarction	17.7	23.4	<0.001
Atrial fibrillation/flutter	30.2	30.9	0.59
Cerebrovascular disease	11.6	12.2	0.50
Hypertension	51.8	42.3	<0.001
Peripheral vascular disease	5.0	9.1	<0.001
Valvular heart disease	17.6	14.4	0.001
Other comorbidities, %			
Cancer, metastatic	2.2	2.0	0.67
Cancer, primary	4.9	6.6	0.011
Dementia	4.9	5.9	0.093
Diabetes	23.5	20.0	<0.001
Diabetes with chronic complications	14.0	10.1	<0.001
Digestive ulcer	3.5	2.6	0.030
Chronic obstructive pulmonary disease	13.4	22.3	<0.001
Rheumatologic diseases	1.0	2.1	0.006
HF etiology, %			
Ischemic heart disease	35.3	47.7	<0.001
Valvular heart disease	11.6	7.2	
Hypertension	26.1	16.1	
Unknown/other	27.1	28.9	

Among cardiovascular comorbidities, a greater proportion of non-Chinese patients had been previously diagnosed with acute myocardial infarction (23.4% vs. 17.7%, p < 0.001) and peripheral vascular disease (9.1% vs. 5.0%, p < 0.001) (Table 1). Conversely, Chinese patients displayed a higher prevalence of valvular heart disease (17.6% vs. 14.4%, p = 0.001) and hypertension (51.8% and 42.3%, p < 0.001).

The two groups also exhibited differences in the distribution of HF etiology. HF was associated with ischemic heart disease in a greater proportion of non-Chinese patients than Chinese patients (47.7% vs. 35.3%, p < 0.001) (Table 1). A larger percentage of Chinese patients experienced HF caused by valvular heart disease (11.6% vs. 7.2% p < 0.001) or hypertension (26.1% vs. 16.1%, p < 0.001).

### Pharmacologic management

Rates of HF medications prescribed within 90 days of discharge were determined for all patients 65 years of age or over. Chinese patients were less frequently prescribed ACE inhibitors (57.5% vs. 66.4%, p < 0.001) but more frequently prescribed ARBs (17.4% vs. 8.9%, p < 0.001) compared to non-Chinese patients (Table 2). A greater proportion of Chinese patients was prescribed both ACE inhibitors and ARBs (6.1% vs. 2.9%, p < 0.001) within 90 days of discharge. The tendency to prescribe either ACE inhibitors or ARBs was higher among non-Chinese patients (72.4% vs. 68.8%, p = 0.011). In addition, Chinese patients were less frequently prescribed digoxin, furosemide, and spironolactone but were more frequently prescribed second-generation calcium channel blockers. Prescribing rates for beta-blockers were comparable between the two groups.

**Table 2:** Rates of medications prescribed within 90 days of discharge

	**Chinese (n = 1,004)**	**Non-Chinese (n = 73,708)**	***P***
Medications prescribed (%)			
ACE inhibitors	57.5	66.4	<0.001
ARBs	17.4	8.9	<0.001
ARBs and ACE inhibitors	6.1	2.9	<0.001
ARBs or ACE inhibitors	68.8	72.4	0.011
Amiodarone	9.1	8.3	0.37
Beta-blockers	42.5	41.6	0.54
Calcium channel blockers, 1st generation	14.9	14.2	0.48
Calcium channel blockers, 2nd generation	21.6	15.3	<0.001
COX-2 inhibitors	3.2	2.8	0.48
Digoxin	29.4	36.1	<0.001
Furosemide	81.5	85.6	<0.001
NSAIDs (excluding COX-2 inhibitors)	5.0	5.7	0.36
Spironolactone	12.6	15.0	0.033
Statins	27.6	25.9	0.22
Vasodilators	42.6	42.9	0.87
Warfarin	27.6	28.3	0.62

Rates of medication prescribed up to 90 days prior to the index admission were also examined to determine whether the differences in use of ACE inhibitors and ARBs after discharge existed before hospitalization. Chinese patients were less frequently prescribed ACE inhibitors (35.7% vs. 39.6%, p = 0.011) and more frequently prescribed ARBs (14.8% vs. 7.6%, p < 0.001) before the first episode of HF requiring hospitalization (Table 3). Chinese patients were also more likely to have been prescribed both an ACE inhibitor and an ARB within the 90-days prior to the index event (2.9% vs. 1.6%, p < 0.001), a trend that persisted after hospitalization.

**Table 3:** Rates of medications prescribed 90 days prior to index admission

	**Chinese (n = 1,004)**	**Non-Chinese (n = 73,708)**	***P***
Medications prescribed (%)			
ACE inhibitors	35.7	39.6	0.011
ARBs	14.8	7.6	<0.001
ARBs and ACE inhibitors	2.9	1.6	<0.001
ARBs or ACE inhibitors	47.6	45.7	0.22

We determined the percentage of patients prescribed ACE inhibitors, ARBs, or beta-blockers within 30-days of discharge who achieved high long-term adherence to these medications (Table 4). Compared to Chinese patients, non-Chinese patients demonstrated higher adherence to ACE inhibitors over the one-year period following the time when the first post-discharge prescription was filled (76.7% vs. 67.6%, p < 0.001). In contrast, high adherence to ARBs was more common among Chinese patients, though this difference was not statistically significant (79.0% vs. 76.0%, p = 0.44). Beta-blocker adherence after one year was similar between the two different groups.

**Table 4:** Proportion of patients displaying high adherence* to medications after discharge

	**High adherence within 6 months of filling the first prescription**			**High adherence within 1 year of filling the first prescription**		
	**Chinese**	**Non-Chinese**	***P***	**Chinese**	**Non-Chinese**	***P***
	**n(%)**	**n(%)**		**n(%)**	**n(%)**	
Patients prescribed medication within 30 days of discharge						
ACE inhibitors	286 (74.7)	25,328 (82.4)	<0.001	259 (67.6)	23,568 (76.7)	<0.001
ARBs	102 (82.3)	3428 (81.4)	0.81	98 (79.0)	3201 (76.0)	0.44
Beta blockers	252 (78.5)	17,208 (81.3)	0.21	242 (75.4)	16,319 (77.1)	0.48

*High adherence is defined as having prescriptions filled to cover at least 80% of days during the 6-month and 1-year periods following discharge.

We conducted a sensitivity analysis in which we excluded patients who were prescribed these medications up to 90 days prior to the index admission in order to assess adherence among new users of the medications. The results of this analysis were similar to the overall adherence analysis, however, the gap in adherence rates between Chinese and non-Chinese patients for ACE inhibitors and ARBs increased (data not shown).

### Outcomes

Chinese and non-Chinese patients displayed comparable crude case-fatality rates at 30 days (12.3% vs. 10.9%, p = 0.10) and at 1 year (31.7% vs. 30.2%, p = 0.24) following the index admission (Table 5). Among patients who survived to discharge, little difference was observed in the crude rate of readmission for HF at 30 days (5.2% in Chinese patients vs. 6.0% in non-Chinese patients, p = 0.23). However, Chinese patients were readmitted noticeably less frequently for HF at 1 year (16.6% vs. 19.4%, p = 0.014). Nevertheless, such a difference between Chinese and non-Chinese patients did not carry forward to the crude rates of readmission for any reason at 30 days (16.2% vs. 16.5%, p = 0.80) and at 1 year (50.0% vs. 51.5%, p = 0.28). Overall, Chinese and non-Chinese patients exhibited a similar event rate of either death or readmission for any reason at 30 days (16.6% vs. 16.0%, p = 0.58) and at 1 year (41.4% vs. 42.0%, p = 0.65).

**Table 5:** Case-fatality and readmission rates

	**Chinese**	**Non-Chinese**	***P***
**Case-fatality rate, %**			
n, all patients	1,339	97,939	
At 30 days			
Crude, %	12.3	10.9	0.10
Risk-adjusted, % (95% CI)	12.3 (10.7-13.9)	10.9 (10.7-11.0)	-
At 1 year			
Crude, %	31.7	30.2	0.24
Risk-adjusted, % (95% CI)	31.3 (29.1-33.6)	30.2 (29.9-30.5)	-
**Readmission rate, %**			
n, discharged alive	1,211	89,022	
For HF			
At 30 days			
Crude, %	5.2	6.0	0.23
Risk-adjusted, % (95% CI)	5.1 (3.8-6.4)	6.0 (5.9-6.2)	-
At 1 year			
Crude, %	16.6	19.4	0.014
Risk-adjusted, % (95% CI)	16.4 (14.2-18.6)^†^	19.4 (19.2-19.7)	-
For any reason			
At 30 days			
Crude, %	16.2	16.5	0.80
Risk-adjusted, % (95% CI)	16.1 (14.1-18.2)	16.5 (16.2-16.7)	-
At 1 year			
Crude, %	50.0	51.5	0.28
Risk-adjusted, % (95% CI)	49.8 (47.0-52.5)	51.5 (51.2-51.9)	-
Composite rate, %*			
n, all patients	1339	97,939	
At 30 days			
Crude, %	16.6	16.0	0.58
Risk-adjusted, % (95% CI)	16.2 (14.3-18.1)	16.0 (15.8-16.2)	-
At 1 year			
Crude, %	41.4	42.0	0.65
Risk-adjusted, % (95% CI)	41.0 (38.5-43.5)	42.0 (41.7-42.3)	-

*Composite rate defined as the rate of death or readmission for heart failure.

^†^ Statistically significantly lower than the overall rate.

Similar trends were observed after risk-adjustment. The one-year risk-adjusted rate of readmission for HF among Chinese patients (16.4%; 95% CI: 14.2%-18.6%) was statistically significantly lower than the overall crude rate for all patients. The risk-adjusted rates for the other outcomes were not significantly different from the overall rate.

### Immigration status analysis

Through linkage to the Canadian Permanent Resident Database, we identified that approximately 30% (n = 391) of the Chinese heart failure patients were relatively recent immigrants to Ontario having immigrated after 1984, as compared with only 3% (n = 3,640) of the non-Chinese patients in the study cohort. A majority of the recent Chinese immigrants came from China (71%) while 12% came from Hong Kong and the rest from other countries. A sensitivity analysis showed that recent Chinese immigrants were slightly younger than longer-term Chinese residents of Ontario (75 vs 77 years mean age), but otherwise, the causes of heart failure, medical management, and long-term outcomes were similar between the two groups (data not shown but available upon request).

## Discussion

In the current study of Chinese and non-Chinese patients with an index hospitalization for HF, we found significant differences in HF etiology and pharmacotherapy. Among Chinese patients, HF was less commonly associated with ischemic heart disease and more commonly due to hypertension and valvular etiologies. Chinese patients were less frequently prescribed ACE inhibitors and more frequently prescribed ARBs. In addition, Chinese patients who were prescribed ACE inhibitors were less adherent to ACE inhibitors over a 1-year follow-up period. Despite these differences, 1-year mortality outcomes between the Chinese and non-Chinese patients were similar.

The finding that ischemic heart disease was a more common cause of HF in non-Chinese patients compared to Chinese patients may be a reflection of the consistently lower rate of ischemic heart disease observed among Chinese individuals as compared to other ethnic groups living in Canada or the United States [3,12,13]. Hypertension and valvular heart disease were relatively more common sources of HF in Chinese patients than non-Chinese patients [14,15]. Older Chinese immigrants to Canada may not have had access to good hypertension and/or rheumatic heart disease screening and treatment in China. Previous studies from Asia have also pointed to hypertension as the most important risk factor among Chinese patients suffering from HF [16,17]. Our finding that the causes of heart failure were similar, regardless of whether a Chinese HF patient was a recent immigrant or longer-term resident of Ontario, suggests that there are true biological differences between Chinese and non-Chinese heart failure patients.

The higher rate of hypertension along with the lower rate of ischemic heart disease may signify a greater prevalence of HF with preserved systolic function among Chinese patients. Patients with preserved ejection fraction are more likely to have hypertension and less likely to have ischemic heart disease than patients with reduced ejection fraction [11,18,19]. In a tertiary care cardiology clinic in Toronto, a greater proportion of Chinese patients than white patients with HF were indeed found to have a preserved ejection fraction [3]. A study of Chinese patients in Hong Kong similarly showed that HF with normal systolic function was more common than heart failure due to systolic dysfunction [20]. Unfortunately, our study was limited by the lack of data on ejection fraction.

In our current study, we found that a lower proportion of Chinese patients were prescribed ACE inhibitors both before hospital admission and after discharge. A high incidence of cough has been reported in Chinese patients on ACE inhibitors in studies from Asia [21,22] though one study has refuted this claim [23]. The higher proportion of Chinese patients who were prescribed ARBs, or both ARBs and ACE inhibitors, could be due to actual intolerance to ACE inhibitors or a general perception among physicians that Chinese patients will be more likely to experience an associated cough. The lower adherence to ACE inhibitors observed among the Chinese patients supports the former hypothesis.

Rates of case-fatality and readmission were similar between the two groups. One exception was the lower 1-year rate of readmission for HF among Chinese patients despite the comparable case-fatality rates and rates of readmission for any reason. The reasons for this finding are uncertain from this study and warrant further investigation. The outcomes in this study stand in contrast to a study conducted using administrative databases from Alberta in which Chinese HF patients had a higher adjusted 1-year mortality rate than White or South Asian patients [5]. The authors of that study could not identify the reason for their observation but the differences between these two studies could reflect differences in the types of Chinese HF patients in the two provinces with HF patients of Chinese descent in Alberta being sicker than those in Ontario. COPD rates, a reflection of smoking rates, were noticeably higher in Chinese relative to non-Chinese patients in Alberta whereas the reverse was noted in the Ontario data. Other factors (e.g. socioeconomic status, access to health care, time since immigration) may also contribute to the disparate findings and warrant further investigation.

Our study has several limitations. First, we identified patients as Chinese or non-Chinese based on a validated list of Chinese surnames rather than self-reported ethnicity, and some participants may have been misclassified. Second, we could not identify from the linked administrative databases whether the participants had preserved or reduced ejection fraction, and we had limited data regarding other patient characteristics and in-hospital HF management as would be contained in a heart failure clinical registry. Third, we could not identify why Chinese patients were less likely to adhere to ACE inhibitors although it was likely due in part to cough and intolerance. Despite these limitations, this is one of largest studies of the real-world outcomes of HF patients of Chinese descent conducted outside of Asia, and provides important comparative information that may be of use to clinicians treating such patients in various clinical settings around the world.

## Conclusion

In conclusion, there are significant differences in HF etiology and the medications used to manage HF between Chinese and non-Chinese patients experiencing their first HF event requiring hospitalization. Future research efforts should investigate why these differences exist and what strategies could improve outcomes for patients with HF in different ethnic groups.

## Competing interests

None of the authors have financial or non-financial competing interests.

## Authors’ contributions

DFY and JVT conceived the idea for the manuscript and co-wrote the first draft. All authors provided input into analysis and interpretation of the data and revising it critically for important intellectual content. All authors approved the final version of the manuscript. JVT and CL obtained funding for the study.

## Pre-publication history

The pre-publication history for this paper can be accessed here:

http://www.biomedcentral.com/1471-2261/13/114/prepub

## Supplementary Material

Additional file 1 Heart failure risk-adjustment model covariates. Click here for file
