# Supplementary material for: A comparison of Chinese and non-Chinese Canadian patients hospitalized with heart failure
Source: BMC Cardiovasc Disord. 2013 Dec 10;13:114. doi: 10.1186/1471-2261-13-114 (PMC4029301; doi:10.1186/1471-2261-13-114)
Supplement: Additional file 1 — Heart failure risk-adjustment model covariates. [file 1471-2261-13-114-S1.doc]

Additional file 1- Heart failure risk-adjustment model covariates

| **Case-fatality model covariates[12]** | **Readmission model covariates[13]** |
| --- | --- |
| Age, years over 65 | Age, years over 65 |
| Male | Male |
| History of percutaneous transluminal coronary angioplasty | History of coronary artery bypass graft surgery |
| History of coronary artery bypass graft surgery | Acute coronary syndrome |
| History of myocardial infarction | Arrhythmias |
| Unstable angina | Chronic atherosclerosis |
| Chronic atherosclerosis | Cardiorespiratory failure and shock |
| Cardiopulmonary-respiratory failure and shock | Valvular and rheumatic heart disease |
| Valvular heart disease | Vascular or circulatory disease |
| Hypertension | Other and unspecified heart disease |
| Stroke | Stroke |
| Renal failure | Renal failure |
| Chronic obstructive pulmonary disease | Chronic obstructive pulmonary disease |
| Pneumonia | Pneumonia |
| Diabetes | Diabetes and diabetes mellitus complications |
| Protein-calorie malnutrition | Protein-calorie malnutrition |
| Dementia | Dementia and senility |
| Hemiplegia, paraplegia, paralysis, functional disability | Hemiplegia, paraplegia, paralysis, functional disability |
| Peripheral vascular disease | Metastatic cancer and acute leukemia |
| Metastatic cancer | Cancer (non-metastatic) |
| Trauma in last year | Major psychiatric disorders |
| Major psychiatric disorders | Other psychiatric disorders |
| Chronic liver disease | Depression |
|  | Liver and biliary disease |
|  | End-stage renal disease or dialysis |
|  | Disorders of fluid/electrolyte/acid–base |
|  | Other urinary tract disorders |
|  | Decubitus ulcer or chronic skin ulcer |
|  | Other gastrointestinal disorders |
|  | Peptic ulcer, hemorrhage, other specified gastrointestinal disorders |
|  | Severe hematologic disorders |
|  | Nephritis |
|  | Asthma |
|  | Drug/alcohol abuse/dependence/psychosis |
|  | Fibrosis of lung and other chronic lung disorders |
|  | Iron deficiency and other/unspecified anemias and blood disease |
